# GMIP: A Novel Prognostic Biomarker Influencing Immune Infiltration and Tumour Dynamics Across Cancer Types

**DOI:** 10.1111/jcmm.70476

**Published:** 2025-04-24

**Authors:** Chao Jiang, Ningfeng Zhou, Xin Xu, Aochen Lv, Shenren Chang, Jiajie Wu, Xiang Li, Aijun Sun, Shiyan Wang, WenZe Tian

**Affiliations:** ^1^ Department of Oncology The Affiliated Huaian No. 1 People's Hospital of Nanjing Medical University Huaian China; ^2^ Department of Spinal Surgery Shanghai East Hospital, School of Medicine, Tongji University Shanghai China; ^3^ Department of Thyroid and Breast Oncological Surgery Huai'an Second People's Hospital, The Affiliated Huaian Hospital of Xuzhou Medical University Huaian Jiangsu China; ^4^ Huaiyin Institute of Technology Huaian Jiangsu China; ^5^ Department of Thoracic Surgery The Affiliated Huaian No. 1 People's Hospital of Nanjing Medical University Huaian China

**Keywords:** GMIP, immunotherapy drug susceptibility, pan‐cancer, prognosis, tumour immunity

## Abstract

GMIP, a member of the RhoGAP family, plays a critical role in cytoskeletal remodelling, cell migration and immune modulation. Its aberrant expression in cancers suggests a pivotal role in tumour progression. GMIP expression was assessed using transcriptomic datasets from GDC and UCSC XENA, and protein distribution across tissues via HPA and GeneMANIA. The TISCH database identified primary GMIP‐expressing cell types in the tumour microenvironment. Univariate Cox regression assessed GMIP's prognostic potential, while cBioPortal and GSCA explored genomic alterations. TIMER 2.0 was used to investigate immune cell infiltration and GMIP's role in immune regulation. GSEA and GSVA unveiled GMIP‐related biological pathways, and molecular docking with CellMiner identified potential drug interactions. In vitro assays confirmed GMIP's functional relevance in breast cancer. GMIP exhibits differential expression across multiple cancer types, demonstrating significant prognostic implications. Its expression is inversely correlated with CNV and methylation in several cancers. GMIP is closely linked to immunotherapy biomarkers and immune suppression, influencing therapeutic responses. Functional studies suggest that GMIP inhibition reduces cancer cell proliferation and migration. GMIP is identified as a promising oncological biomarker, particularly in breast cancer, with potential therapeutic implications. GMIP's therapeutic potential is especially pronounced in BRCA‐mutated tumours, underscoring its relevance for novel anticancer interventions.

## 1 Introduction

Cancer is a multifaceted disease characterised by abnormal cell proliferation, invasion and metastasis, orchestrated by genetic, epigenetic and molecular aberrations. As the second leading cause of death globally, cancer diagnosis, prognosis and treatment remain challenging. The rapid advancement of high‐throughput sequencing has ushered in a new era of cancer research, providing vast genomic, transcriptomic, proteomic and epigenomic data that enhance our understanding of cancer mechanisms [1, 2]. In this context, comprehensive bioinformatics analyses have become essential for uncovering the molecular drivers of cancer progression and therapeutic resistance. Immune checkpoint inhibitors, such as CTLA‐4 and PD‐1 blockers, have significantly advanced cancer immunotherapy [3]. However, many patients exhibit resistance and fail to achieve durable responses. Thus, identifying novel immunotherapy biomarkers or immune‐modulatory genes is crucial for developing personalised treatments and improving long‐term outcomes [4]. The advent of bioinformatics and the expansion of public databases have facilitated the systematic identification of therapeutic targets through integrative multi‐omics analyses [5, 6].Unlike traditional laboratory‐based screening, bioinformatics‐driven approaches offer distinct advantages: high‐throughput candidate identification, multidimensional validation integrating genomic, transcriptomic and clinical data, and reduced experimental costs and time investment. Through such comprehensive bioinformatics analysis workflows, researchers have discovered several promising biomarkers with pan‐cancer prognostic value, which have demonstrated robust predictive capabilities across various cancer types. These findings not only validate the effectiveness of bioinformatics‐based target discovery but also provide new perspectives for the development of targeted therapies, immunotherapies and combination treatments.

GMIP (GEM interacting protein) encodes a protein involved in GTPase‐activating proteins (GAPs) (GENE ID: 51291). Its primary function is to regulate the Rho GTPase signalling pathway, which is key to processes like cytoskeletal remodelling, cell migration and proliferation, all closely tied to cancer development [7, 8, 9, 10]. GMIP interacts with Gem via its N‐terminal domain and contains a cysteine‐rich domain, followed by a RhoGAP domain at the C‐terminus. The RhoGAP domain enhances RhoA GTPase activity in vitro, and through its interaction with GEM, GMIP regulates RhoA activity, influencing cell movement and morphology [11]. GMIP plays a key role in regulating the stability and processing of RNA, participates in various RNA metabolic pathways, and is an overall regulator of gene expression and cellular function. These functions are essential for maintaining the normal operations of the cell.

Exploring gene function is a critical method in biomedical research for understanding disease mechanisms and identifying potential therapeutic targets. GMIP belongs to the ARHGAP family of genes, and its family member DLC1 has been reported to be highly associated with cancer [12, 13]. Recent studies have predicted a correlation between GMIP and endometrial cancer [14], suggesting the potential of GMIP as a novel cancer marker. As a GTPase‐activating protein (GAP) for RhoA, GMIP can promote the hydrolysis of GTP by RhoA, converting it from an active GTP‐bound state to an inactive GDP‐bound state. RhoA is crucial for regulating the cytoskeleton, cell migration and adhesion, and GMIP significantly influences cell structure and movement by modulating RhoA activity [15]. Through RhoA regulation, GMIP impacts cytoskeletal stability and reorganisation, affecting cell morphology, migration, division and adhesion—key processes in tissue development, wound healing and cancer metastasis. Since cell migration is critical for metastasis, GMIP's regulation of RhoA is particularly important for cancer cell migration. Although research on GMIP remains limited, studies suggest its potential role in cancer [16]. Abnormal GMIP expression can disrupt RhoA signalling, thereby influencing cell migration, invasion and proliferation [17]. In endometrial cancer, GMIP's differential expression has been confirmed via immunohistochemistry and Western blot, with upregulation correlating with increased tumour invasiveness and metastasis [14]. Thus, GMIP may act as an oncogene in some cancers, though its role may vary and requires further investigation.

Currently, most studies on the relationship between GMIP and cancer focus on specific cancer types [18, 19], while its role across various cancers remains underexplored. This article reviews the progress of GMIP research in different tumours. We systematically analysed GMIP expression and its prognostic impact in various malignancies using databases such as TCGA, HPA, CCLE, GTEx and TISCH. Additionally, we conducted a genome‐wide analysis to assess GMIP's associations with DNA methylation, copy number variation (CNV), tumour mutation burden (TMB), microsatellite instability (MSI) and immune infiltration. To explore GMIP's role in 33 cancers, we analysed its co‐expression with stromal, immune and ESTIMATE scores, mismatch repair (MMR) markers and immune‐related genes. Furthermore, we used molecular docking to evaluate GMIP's three‐dimensional structure and tested its sensitivity to GSEA, GSVA and various anticancer drugs.

Our findings highlight GMIP's pivotal role in breast cancer cell survival and migration. We investigated its oncogenic mechanisms across various cancers, along with its prognostic value and immune‐related associations. By comparing GMIP expression in tumour and normal tissues, we evaluated its distribution, prognostic impact and links to genomic stability and immune cell infiltration. The results underscore GMIP's significance in cancer initiation and progression, providing a foundation for future research on its role in tumour immunity and its potential as a target for novel immunotherapies.

## 2 Materials and Methods

### 2.1 Public Data and Analysis Methods

A disease‐phenotype association analysis for GMIP was conducted using the Open Targets platform (URL: https://platform.opentargets.org/) [20], visualised via bubble charts, and the subcellular localization of the GMIP protein was validated using the Human Protein Atlas (HPA) database (URL: https://www.proteinatlas.org/) [21].The subcellular distribution of the GMIP protein was analysed. RNA‐seq data (TPM format) from UCSC XENA were analysed to assess GMIP's differential expression across 33 cancer types and matched normal tissues. The data were processed through standardised workflows to ensure consistency. By analysing the expression levels of GMIP in these cancers and their corresponding normal tissues, we assessed its differential expression, with the data standardised to ensure consistency. The Gene_DE module of the TIMER2.0 platform was used to analyse the mRNA expression differences of GMIP between cancer and adjacent normal tissues [22]. Additionally, expression data from 29 cancer cell lines were collected from the Cancer Cell Line Encyclopedia (CCLE) database (URL: https://portals.broadinstitute.org/ccle/) [23]. Apart from differential expression, all subsequent analyses used mRNA expression in TPM format, and clinical data were derived from the TCGA dataset, which was obtained through the GDC data portal. Furthermore, we constructed a protein‐protein interaction (PPI) network using GeneMANIA (http://www.genemania.org) to obtain interaction information for GMIP, predict its function, and identify functionally similar genes (e.g., physical interactions, co‐expression, co‐localization, genetic interactions, etc.). Meanwhile, we also analysed data from two immunotherapy cohorts. The IMvigor210 cohort (data sourced from http://research‐pub.gene.com/IMvigor210CoreBiologies/packageVersions/) included 298 urothelial cancer patients treated with Atezolizumab (anti‐PD‐L1), while the GSE91061 cohort (data from the GEO database: https://www.ncbi.nlm.nih.gov/geo/) included 51 melanoma patients treated with Nivolumab (anti‐PD‐1). For specific information on cancer abbreviations, see Table S1.

### 2.2 Single‐Cell Analysis and Spatially Resolved Transcriptomics

Using the TISCH tool (URL: http://tisch1.comp‐genomics.org/) for single‐cell analysis, we included the GMIP gene, major cell lines and all cancer types as parameters. The expression of GMIP across different cell types is visualised through a heatmap. Additionally, leveraging the STOmics DB database, GMIP shows significant spatial overlap with the CD8+ T cell markers CD8A and CD8B in LIHC tumour tissues. Similarly, in NSCLC tumour tissues, GMIP exhibits a strong spatial correlation with the M2 macrophage markers CD163 and CD68.

### 2.3 Survival and Prognosis Analysis

Cox regression analysis was performed using the PanCanSurvPlot platform (URL: https://smuonco.shinyapps.io/PanCanSurvPlot/) to evaluate the association between GMIP expression and overall survival (OS), disease‐specific survival (DSS), disease‐free interval (DFI) and progression‐free survival (PFS). The analysis used data from the TCGA database, which was based on the IlluminaHiSeq platform and employed the best cut‐off grouping method [24]. Results were presented using the ‘forestplot’ R package, expressed as hazard ratios (HR) with 95% confidence intervals (95% CI).

### 2.4 Genomic Alterations in Cancer and GMIP Mutation Overview

The cancer type summary module in cBioPortal (URL: https://www.cbioportal.org/) was used to analyse the frequencies of four types of genomic alterations (mutations, amplifications, deep deletions and multiple alterations) in tumours. Meanwhile, the GSCA platform (URL: http://bioinfo.life.hust.edu.cn/GSCA) was used to investigate the distribution of GMIP gene CNVs [25], the relationship between GMIP mRNA expression and CNVs, and the impact of different CNVs on GMIP. Furthermore, the GSCA platform was used to analyse the differences in GMIP methylation levels across different cancers and to evaluate the correlation between GMIP mRNA expression and methylation levels, comparing survival differences between high and low methylation groups. Spearman correlation analysis was conducted to assess the relationship between GMIP mRNA expression and CNV or methylation levels, with *p*‐values adjusted using FDR. Differences in methylation were estimated using *t*‐tests, and the *p*‐values were further corrected by FDR. Survival analysis was performed using the log‐rank test to evaluate differences in OS, DSS and PFS between the two groups.

### 2.5 Immunotherapy Predictive Analysis

Somatic mutation data was obtained from the TCGA database (URL: https://tcga.xenahubs.net), and the tumour mutation burden (TMB) and microsatellite instability (MSI) for each tumour sample were calculated using the R package ‘maftools.’ Subsequently, the associations between GMIP gene expression, TMB and MSI was assessed using the Spearman rank correlation coefficient, and radar plots were generated for visualisation with the R package ‘gradar.’ In the immunotherapy analysis, treatment responses were categorised into four groups: progressive disease (PD), stable disease (SD), complete response (CR) and partial response (PR). The R package ‘survminer’ was used to determine the optimal cut‐off value for GMIP expression, which was then used to divide the two immunotherapy cohorts into high and low expression groups. Survival and treatment response differences were analysed for each group. Additionally, the expression of MMR genes (including MLH1, MSH2, MSH6, PMS2 and EPCAM) across different cancer types was examined, and their correlations with GMIP were explored. The final results were visualised using heatmaps generated by the R packages ‘tidyverse’ and ‘ggnewscale.’

### 2.6 Evaluation of the Impact of GMIP Expression on Immunity

The ESTIMATE method was used to assess immune and stromal cell infiltration levels in malignant tumour tissues. Using the Illumina platform, the R packages ‘limma’ and ‘ESTIMATE’ were used to analyse the association between GMIP expression and immune score, stromal score and ESTIMATE score [26]. Additionally, the infiltration of immune cells in different cancer types was evaluated, and the relationship between GMIP expression and immune cell infiltration was investigated using the TIMER 2.0 platform. TIMER 2.0 calculates immune cell infiltration scores from the TCGA database; these data were collected and analysed to explore the potential link between GMIP expression and immune infiltration. Furthermore, 150 immune‐related genes were downloaded from TISDB, including those encoding MHC, immune inhibitory factors, chemokine receptors, immune activation factors and chemokine proteins. The R packages ‘limma,’ ‘pheatmap’ and ‘ggplot2’ were then used to analyse the relationship between GMIP expression and these immune genes, with the results visualised accordingly.

### 2.7 Biological Significance Analysis of GMIP Expression

The biological functions of GMIP in tumours were evaluated through GSEA and GSVA analyses. The analysis used the R packages ‘tidyverse,’ ‘limma,’ ‘org.Hs.eg.db,’ ‘gseaplot2’ and ‘clusterProfiler’, and the C2 and C5 gene sets were obtained from the Molecular Signatures Database (MSigDB). The normalised enrichment score (NES) was calculated, and differentially expressed genes between the GMIP high‐ and low‐expression groups across different cancer types were compared, with results corrected using FDR. Samples were divided into high and low expression groups based on the median expression level of GMIP, and GSVA scores were generated for each cancer type. Additionally, to further explore the potential impact of GMIP expression, we integrated multiple R packages, including ‘GSVA,’ ‘ggprism,’ ‘GSEABase,’ ‘ggthemes’ and ‘BiocParallel’ to complete the GSVA analysis, systematically assessing GMIP‐related gene pathways and biological functions across different cancer types.

### 2.8 Correlation Between GMIP Expression and Drug Sensitivity

The CellMiner database (URL: http://discover.nci.nih.gov/cellminer/) revealed a potential association between GMIP expression and drug response [27, 28]. This platform, specifically designed for cancer research, integrates molecular and pharmacological data from the NCI‐60 cancer cell lines [29], which are widely used for anticancer drug screening and research. The processed datasets, including RNA‐seq‐based RNA expression data and drug data related to compound activity in NCI‐60 cell lines, were downloaded from CellMiner; CTRP and GDSC data were accessed from the GSCA website's ‘Drug’ module [30]. The R package ‘limma’ was used to filter FDA‐approved drugs or those in clinical trials, while columns with more than 80% missing values were excluded. For the remaining missing data, the R package ‘Impute’ was used for imputation. Subsequently, the data were visualised using ‘ggplot2’ and ‘ggpubr,’ with *p*‐values less than 0.05 considered significant. Additionally, molecular docking was performed using Autodock4 software to evaluate the binding energy and interaction patterns between docetaxel acetate and the GMIP protein obtained from the CellMiner database. The molecular structure of docetaxel acetate was obtained from the PubChem database (URL: https://pubchem.ncbi.nlm.nih.gov/), while the 3D structure of the GMIP protein was predicted using AlphaFold (URL: https://alphafold.ebi.ac.uk/) [31]. The molecular docking models were visualised using Pymol software.

### 2.9 Cell Culture and Transfection

The human breast cancer cell line, MCF‐7, and the human liver cancer cell line, SK‐Hep‐1, were purchased from the Cell Bank of the Chinese Academy of Sciences (Shanghai, China). The cells were cultured in DMEM medium (Procell, Wuhan, China) supplemented with 10% fetal bovine serum (FBS; Procell). The cells were maintained at 37°C in a 5% CO_2_ incubator, with mycoplasma contamination was regularly monitored by PCR. Anti‐human GMIP siRNA and its negative control were provided by GenePharma (Shanghai, China). According to the manufacturer's instructions, these siRNAs were transfected into the cells using the transfection reagent Lipo3000 (Invitrogen, California, USA). The sequences of the siRNAs are detailed in Table S2.

### 2.10 Reverse Transcription‐Quantitative Polymerase Chain Reaction (RT‐qPCR)

Total RNA was extracted using TRIzol reagent (Takara Bio, Japan), and RNA concentration and purity were evaluated using the NanoDrop 2000 system (Thermo Scientific). Reverse transcription PCR (RT‐PCR) was performed using PrimeScript RT Master Mix (Takara, RR036A), and real‐time quantitative PCR (qPCR) was conducted using SYBR Premix Ex Taq II (Takara, RR820A). Gene expression analysis was conducted using the 2^−∆∆Ct^ method, with ACTB as the internal control. The RT‐qPCR primer sequences are provided in Table S3.

### 2.11 Cell Viability Assay

Cell viability was evaluated using the CCK‐8 kit (GK10001, GLPBIO, Montclair, California, USA). Cells were seeded into 96‐well plates at a density of 2 × 10^3^ cells/well. After 24 or 48 h of incubation, 10 μL of CCK‐8 solution was added to each well. Following a 2‐h incubation, the absorbance at 450 nm was measured using a microplate reader.

### 2.12 Colony Formation

In the colony formation assay, the MCF‐7 cell line and the SK‐Hep‐1 cell line were seeded into 6‐well plates at 1,000 cells per well, with three replicates. The culture medium was refreshed every 3 days. After a 1‐week incubation, the cells were fixed with methanol and stained with 0.5% crystal violet. Colonies were imaged and quantitatively analysed using ImageJ software.

### 2.13 Cell Scratch Wound‐Healing Assay

In the wound healing assay, cells transfected with the specified siRNA were seeded into 6‐well plates at 1 × 10^5^ cells per well, with 2% fetal bovine serum added to the medium to minimise cell proliferation. A pipette tip was used to score the cell monolayer. Images of the wound healing area were captured using a Nikon Ti‐E inverted microscope (Nikon Instruments, Florence, Italy), with images taken at 0 and 48 h post‐scratch. The wound area at each time point was calculated using ImageJ software, and the wound area at each time point was normalised to the initial area at T0.

### 2.14 Statistical Analysis

For bioinformatics validation, the dataset was first filtered to remove missing values and duplicate results, followed by log2 (TPM + 1) transformation of the TPM values. The expression levels of GMIP in normal and tumour tissues were compared using the Mann–Whitney *U* test (Wilcoxon rank‐sum test) to assess statistical significance. For data from different tissue origins in the CCLE database, the Kruskal–Wallis method was used to analyse GMIP expression. Depending on whether the samples were paired, paired *t*‐tests or unpaired *t*‐tests were used to compare heterogeneous GMIP expression levels between different groups or between tumour and normal tissues. A significance level of *p* < 0.05 was set. R software (version 4.4.0; https://www.R‐project.org) was used for all analyses.

## 3 Results

### 3.1 Differential Expression of GMIP and Its Related Genes in Cancer

**FIGURE 1:**
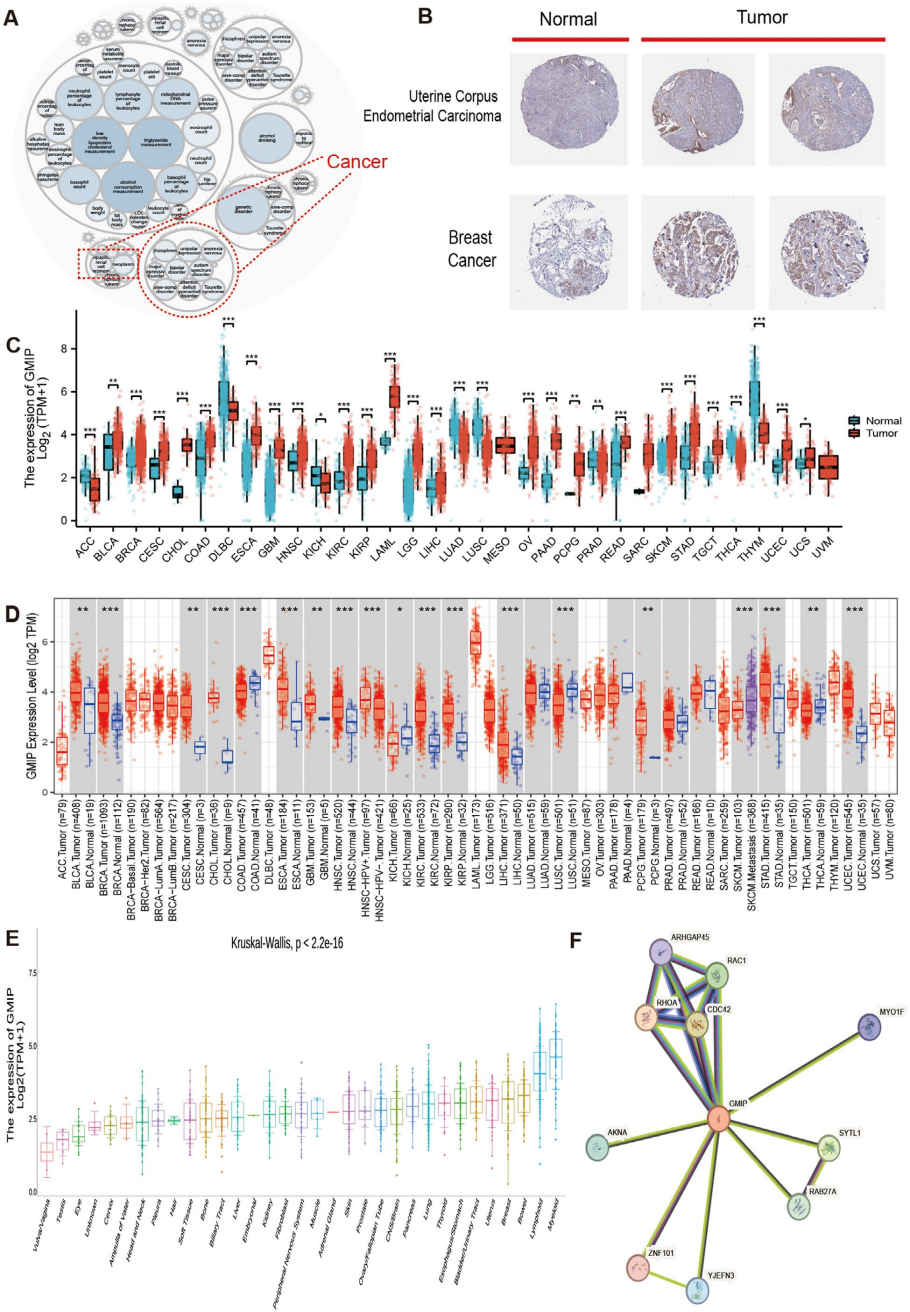
Differential expression of GMIP and its related genes in cancer. (A) The diseases associated with GMIP were analysed on the OpenTarget web tool. The red dashed lines represent GMIP‐associated cancers. (B) A comparison of histological sections between normal tissues and tumour tissues of endometrial cancer and breast cancer. (C) Expression levels of the GMIP gene in different cancer types. (D) The expression status of the GMIP gene in different cancers or specific cancer subtypes was analysed through TIMER2. (E) The expression levels of the GMIP gene in different tissues were statistically significant by Kruskal–Wallis test (*p* < 2.2e‐16). (F) GMIP‐binding proteins obtained by the STRING tool.

GMIP‐associated diseases were identified using Open Targets, with red dashed lines denoting cancer associations (Figure 1A). Figure 1B shows that the immunohistochemical staining of GMIP is at a moderate level in normal liver tissues, while it is significantly enhanced in tumour tissues (Figure S1 illustrates the overall scope of the study). GMIP staining is weak in normal breast tissues but shows a moderate level in tumour tissues. By analysing the TCGA and GTEx databases, we further investigated the transcriptional levels of GMIP in tumours and compared them with normal tissues. Figure 1C indicates that GMIP expression is significantly higher in cancers such as BRCA, CESC, COAD, ESCA, GBM, KIRC, KIRP, LAML, LGG, OV, PAAD, STAD and UCEC compared to normal tissues, whereas its expression is significantly lower in ACC, DLBC, KICH, LUAD, LUSC and THYM. Subsequently, using TIMER2.0, we analysed the differences in GMIP mRNA expression between cancer tissues and adjacent normal tissues. As shown in Figure 1D, GMIP mRNA is significantly upregulated in 13 cancers (e.g., BRCA, BLCA and CESC), while it is significantly downregulated in COAD, KICH and LUSC. Finally, we analysed the expression differences of GMIP in 29 tissue types using publicly available data downloaded from the CCLE database. The results of the Kruskal–Wallis test (*p* < 2.2e‐16) indicate significant differences in GMIP expression levels among different tissues (Figure 1E). In this study, we considered physically binding protein interactions and obtained 10 experimentally supported GMIP‐binding proteins from the STRING network (Figure 1F). Our integration of multiple databases (TCGA and GTEx) provides a more comprehensive and reliable assessment of GMIP expression patterns across diverse cancer types. The expression levels of GMIP across various cancer types were analyzed using data from the TCGA database, and the association between GMIP expression and cancer prognosis was examined (Figure S2).

### 3.2 Single‐Cell Analysis of GMIP in Cancers

**FIGURE 2:**
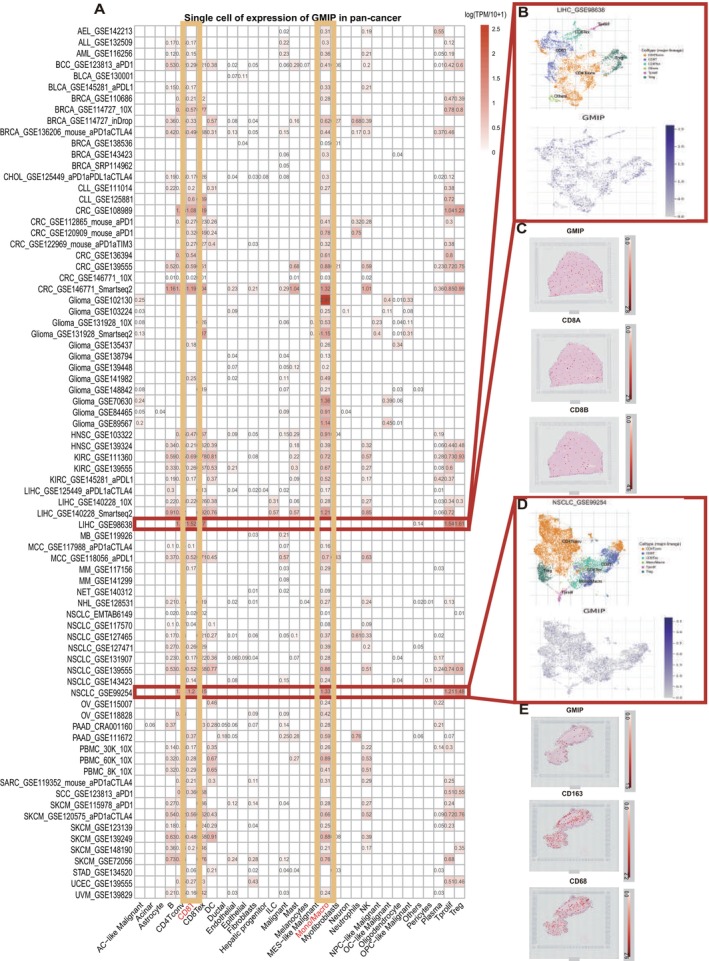
Single‐cell analysis of GMIP in cancers. (A) Summary of GMIP expression of 32 cell types in 78 single‐cell datasets. (B) Scatter plot showed the distributions of different cell types of the LIHC_GSE98638 dataset. (C) Spatial representation of GMIP, CD8A, CD8B. (D) Scatter plot showed the distributions of different cell types of the NSCLC_GSE99254 dataset. (E) Spatial representation of GMIP, CD68 and CD163.

To identify the primary cell types expressing GMIP in the tumour microenvironment (TME), we performed a single‐cell analysis of GMIP across 78 cancer datasets. The heatmap in Figure 2A, generated using the TISCH network tool, illustrates GMIP expression levels across 32 cell types, including both immune cells and stromal cells. The results indicate that GMIP is predominantly expressed in immune cells, especially in CD8+ T cells. In the LIHC_GSE98638 dataset, which includes 5,063 cells from 20 liver cancer patients, the analysis shows that in the LIHC microenvironment, GMIP expression is higher in immune cells, including CD8+ T cells (Figure 2B). Additionally, through single‐cell RNA sequencing analysis of 12,346 single cells from 20 non‐small cell lung cancer (NSCLC) patients in the GSE99254 dataset, we observed that GMIP is primarily expressed in monocytes/macrophages and CD8+ T cells (Figure 2D). Moreover, our spatial transcriptomic analysis demonstrates that in LIHC tumour tissues, GMIP spatially overlaps significantly with the CD8+ T cell markers CD8A and CD8B (Figure 2C). Similarly, in NSCLC tumour tissues, GMIP shows a strong spatial correlation with the M2 macrophage markers CD163 and CD68 (Figure 2E). The similar spatial distribution of these markers suggests that GMIP, CD68, CD163, CD8A and CD8B may be co‐expressed in these cell types.

### 3.3 Genetic Alteration Analysis

**FIGURE 3:**
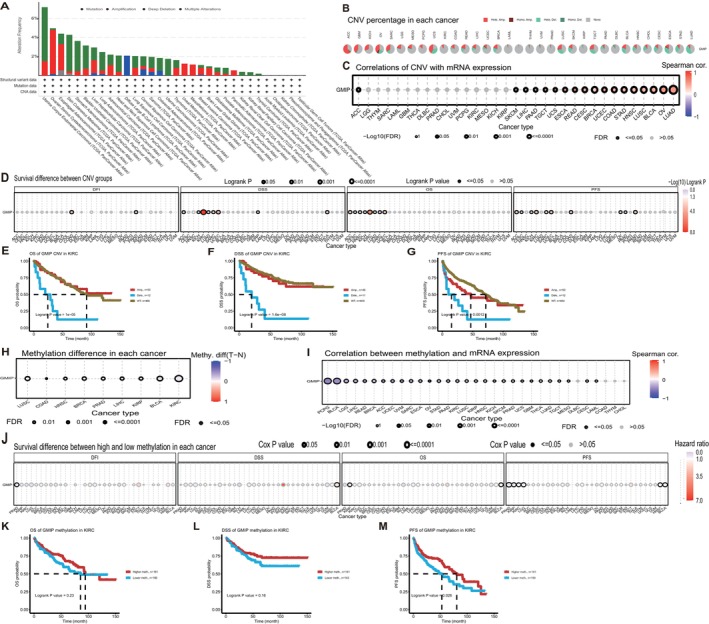
Genetic alteration analysis. (A) shows the variation frequency and types of alterations. (B) and (C) present the CNV correlation analysis. (D) highlights the correlation between CNV and survival outcomes. (E), (F) and (G) display survival curves for KIRC patients. (H) illustrates the differences in GMIP gene methylation across different cancers. (I) shows the methylation correlation analysis. (J) presents the Cox regression analysis used to calculate survival differences between different methylation groups. (K), (L) and (M) demonstrate the impact of GMIP gene methylation levels on three survival outcomes of kidney renal clear cell carcinoma (KIRC) patients.

Genetic alterations in GMIP were examined across TCGA cohorts to assess mutation frequencies and copy number variations in diverse malignancies. As shown in Figure 3A, ‘mutation’ is the most common type of alteration, with the highest rate of GMIP alterations observed in endometrial cancer patients. Figure 3B illustrates the percentage of different types of CNVs (copy number variations) across various cancer types. In the pan‐cancer analysis, the Spearman correlation between GMIP CNV and mRNA expression was assessed. A significant positive correlation between GMIP CNV and mRNA expression was observed in LUAD, OV, BLCA, LUSC, HNSC, STAD, COAD, UCEC, BRCA, CESC, READ, ESCA, UCS, TGCT, PAAD, LIHC and SKCM, whereas a significant negative correlation was found in ACC (Figure 3C). Next, we explored the correlation between GMIP CNV and survival outcomes in different cancer types. The most notable correlation was observed in KIRC (Figure 3D). Subsequently, samples were divided into WT (wild‐type), Amp (amplification) and Dele (deletion) groups, and we investigated the survival differences among CNV and GMIP genotypes in each cancer type. Kaplan–Meier survival curves were used to display the survival probability of patients with different CNV statuses. Specifically, in terms of various survival metrics, patients with high CNV in KIRC consistently had a poor prognosis (Figure 3E–G). DNA methylation is a chemical modification that can lead to the inactivation of tumour suppressor genes and promote carcinogenesis. Figure 3H shows the differential methylation of the GMIP gene across different cancers. In PCPG, BLCA, LGG, LIHC, READ, BRCA, ACC, UCEC, UVM, SARC, ESCA, OV, STAD, PAAD, KIRC, LUSC, KIRP, HNSC, KICH, SKCM, PRAD, UCS, GBM, THCA, LUAD, TGCT, MESO, CESC and LAML, methylation was negatively correlated with GMIP mRNA expression (Figure 3I). Based on the average methylation levels, samples were classified into high‐ and low‐methylation groups. The study found that GMIP acts as a risk factor in KIRC (Figure 3J). Notably, analysis of various survival metrics consistently indicates that KIRC patients with higher GMIP gene methylation levels have a lower risk of death (Figure 3K–M). Our integrated analysis of CNV, mutations and methylation reveals comprehensive genetic regulation mechanisms of GMIP.

### 3.4 Co‐Expression of GMIP With Immune‐Related Genes

**FIGURE 4:**
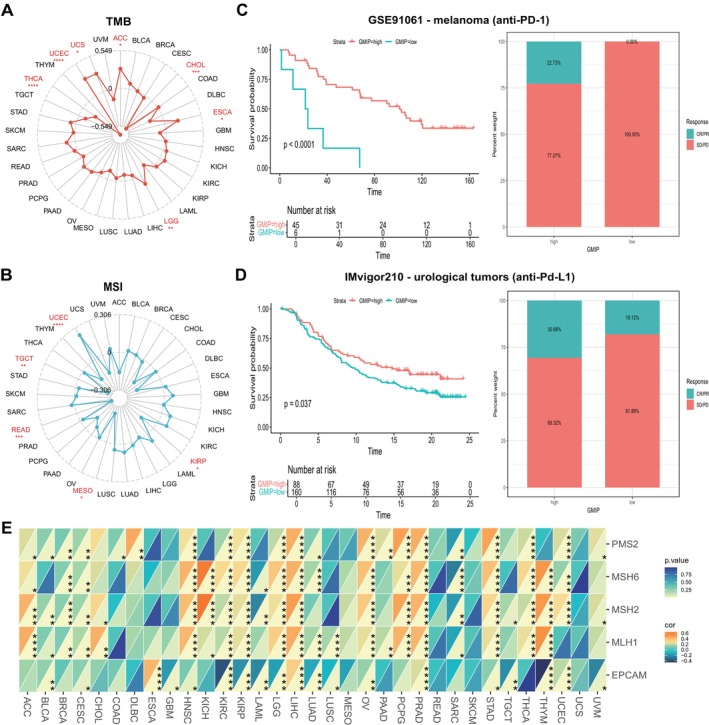
(A) Correlations between GMIP expression and tumour mutation burden in pan‐cancer. (B) Correlations between GMIP expression and microsatellite instability in pan‐cancer. (C) Kaplan–Meier curves for low‐ and high‐GMIP patient groups in GSE91061 (anti‐PD‐L1, melanoma) (D) Kaplan–Meier curves for low‐ and high‐GMIP patient groups in the IMvigor210 cohort (anti‐PD‐L1, urological) (E) Spearman's correlation analysis of GMIP expression with expression levels of five MMR genes across cancers.

We evaluated the correlation between GMIP expression and TMB and MSI. GMIP expression was positively correlated with TMB in ACC, UCS, UCEC, LGG and ESCA (Figure 4A). Additionally, GMIP expression showed a significant positive correlation with MSI in UCEC and a significant negative correlation with MSI in KIRP, MESO, TGCT and READ (Figure 4B). We subsequently analysed the predictive role of GMIP in the ICI cancer cohort. In the GSE91061 melanoma cohort (Figure 4C), melanoma patients with high GMIP expression had significantly better survival rates than those with low expression. The response rate to anti‐PD‐1 treatment in the high GMIP expression group was 22.73%, whereas it was 0% in the low expression group. Additionally, the relationship between GMIP and response to anti‐PD‐L1 therapy in patients with urologic tumours showed that patients with high GMIP expression had better survival rates and longer survival times than those with low expression. The response rate to anti‐PD‐L1 therapy in patients with high GMIP expression was 30.68%, significantly higher than the 18.12% in patients with low GMIP expression. These findings confirm the potential of GMIP as a predictor of immunotherapy response. Furthermore, we evaluated the correlation between GMIP expression levels and mutation rates of five MMR genes. As shown in Figure 4E, in 33 types of cancer, GMIP was highly correlated with MMR gene mutations, except for READ and UCS.

### 3.5 The Correlation Between GMIP and Immune Infiltration Analysis

**FIGURE 5:**
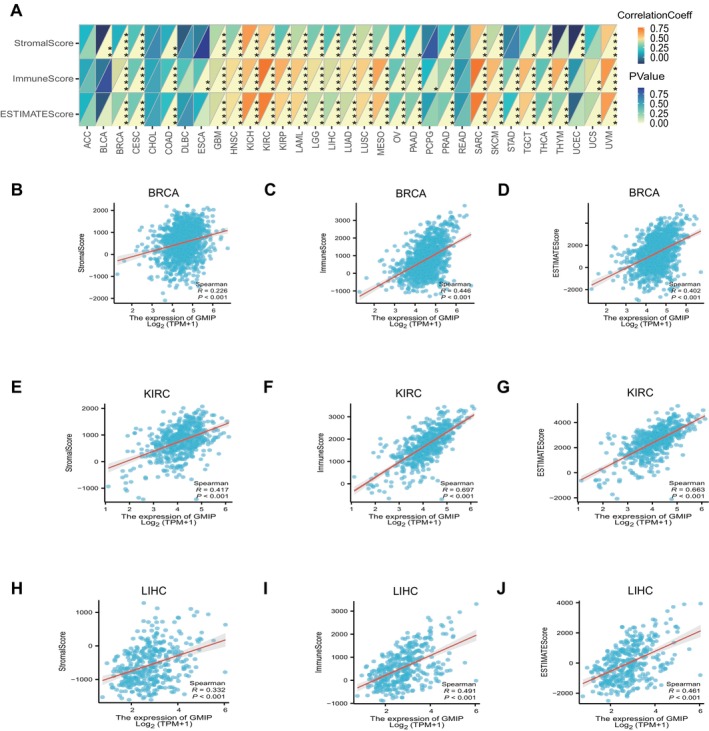
The top three tumours with the most significant correlation between the degree of immune infiltration and GMIP expression were LIHC, KIRC and BRCA (StromalScore); LIHC, KIRC and BRCA (ImmuneScore); LIHC, KIRC and BRCA (ESTIMATEScore), respectively.

The TME is a complex system comprising stromal cells, fibroblasts, endothelial cells, as well as components of both the innate and adaptive immune systems [32, 33]. To explore the connection between GMIP expression and the TME, we comprehensively evaluated the StromalScore, ImmuneScore and ESTIMATE score across different solid tumours (Figure 5A). Our analysis revealed a statistically significant positive correlation between GMIP expression and the StromalScore, ImmuneScore and ESTIMATE score, especially in tumours where these scores were more prominent. Figure 5A clearly illustrates this relationship. The detailed results for BRCA, KIRC and LIHC are shown in Figure 5B–J. These findings suggest that GMIP expression is closely associated with the extent of immune infiltration in tumours. The comprehensive immune infiltration analysis framework could potentially guide immunotherapy strategies by identifying patients most likely to respond to immune checkpoint inhibitors.

### 3.6 Correlation Between GMIP Expression and Immuno‐Permeable/Immunomodulatory Genes

**FIGURE 6:**
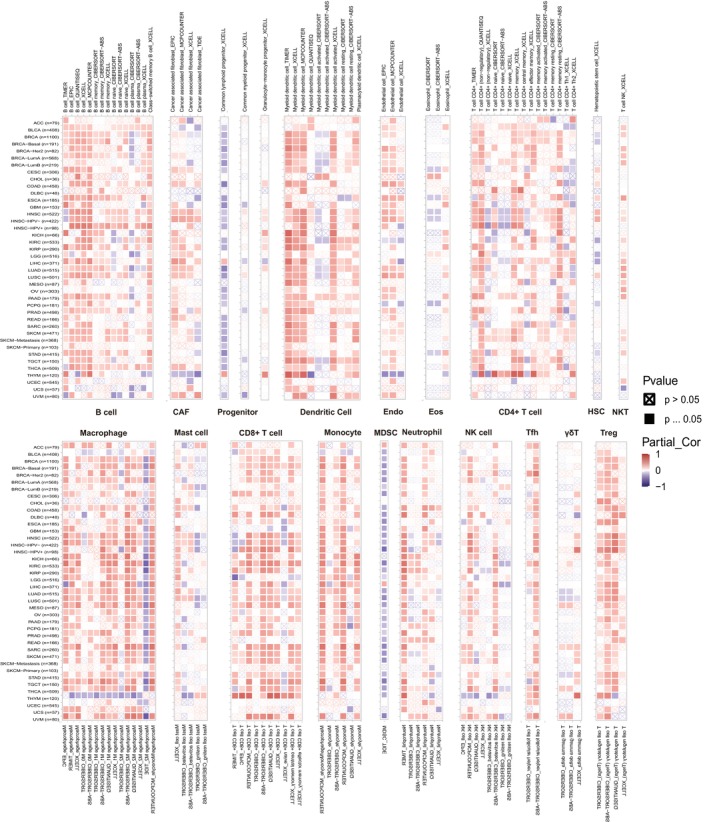
The correlations of GMIP expression and the infiltration levels of CD4+ T cells, CAF, progenitor, Endo, Eos, HSC, Tfh, γ/δ T cells, NKT, regulatory T cells(Tregs), B cells, neutrophils, monocytes, macrophages, dendritic cells, NK cells, Mast cells and CD8+ T cells in cancers. Positive correlation in red and negative correlation in blue.

We further investigated the correlation between GMIP expression and immune cell infiltration. Spearman correlation analysis was performed using pan‐cancer immune cell data from the TIMER2 database. The results showed the infiltration levels of CD4+ T cells, CAF, progenitors of lymphoid, progenitors of myeloid, progenitors of monocytes, Endo, Eos, HSC, Tfh, γ/δ T cells, NKT cells, Tregs, B cells, neutrophils, monocytes, macrophages, dendritic cells, NK cells, mast cells and CD8+ T cells in pan‐cancer tissues. The findings indicated that GMIP expression in most TCGA tumours was positively correlated with the infiltration levels of B cells, CAF, CD4+ T cells and NKT cells, while it was negatively correlated with the infiltration levels of MDSCs. Additionally, particularly in KIRC and BRCA, GMIP expression was positively associated with the infiltration levels of most immune cells, including CD8+ T cells and macrophages. Recent studies have emphasized the critical role of immune cells [34], such as CD4+ T cells, CAFs, MDSCs, neutrophils and macrophages, in tumour immunotherapy, highlighting their undeniable importance in cancer treatment [35, 36]. Our research suggests that GMIP, through its interactions with immune cells, can influence tumorigenesis and holds potential for predicting tumour progression and the effectiveness of antitumour therapies (Figure 6). In addition, the relationship between GMIP expression and immune response genes was examined (Figure S3).

### 3.7 GMIP Expression Biological Significance in Tumours

**FIGURE 7:**
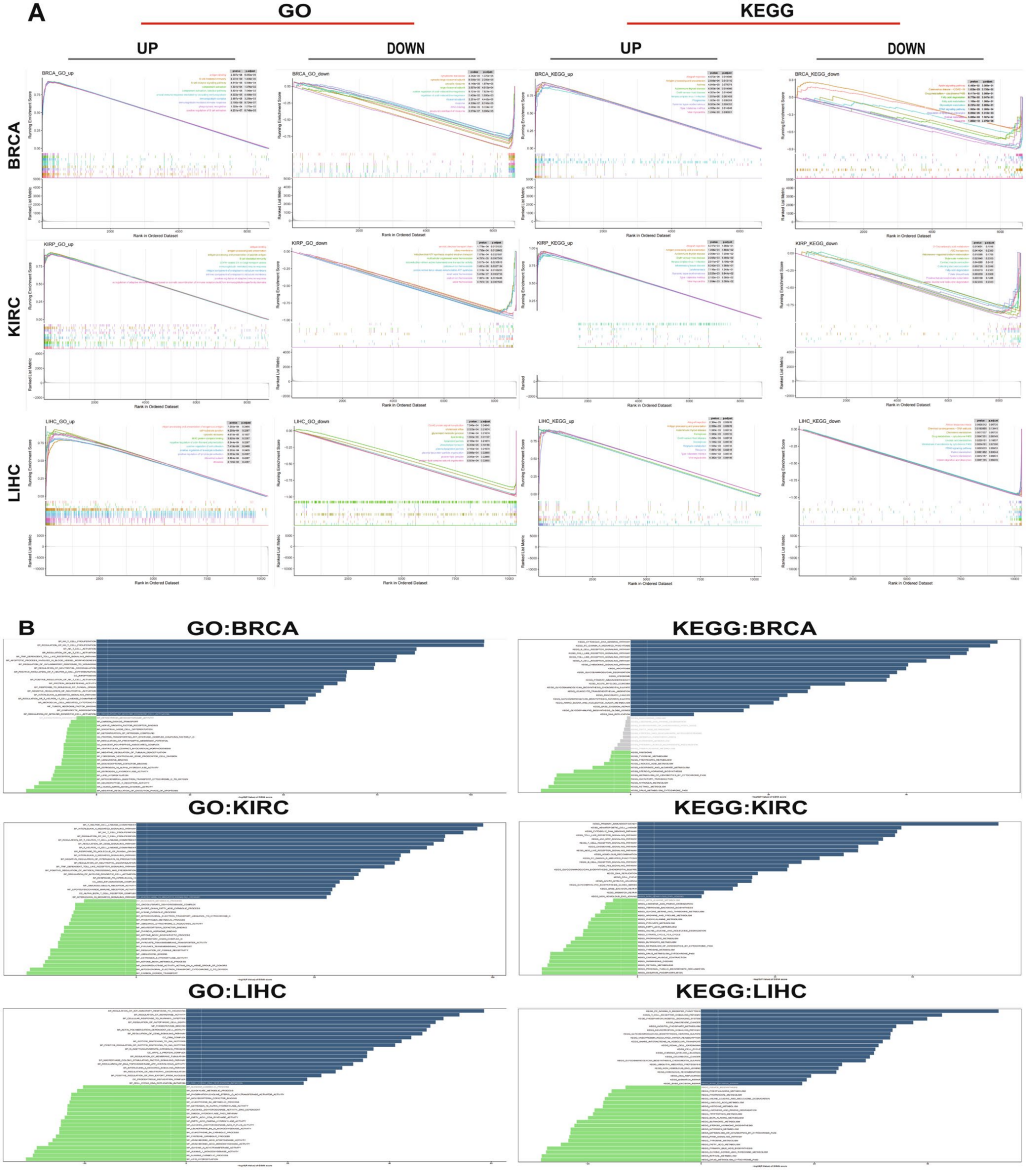
GMIP expression biological significance in tumours (A) The GSEA results of GMIP in BRCA, KIRP and LIHC were validated through GO functional annotation and KEGG pathway analysis. The curves of different colours represent the regulated functions or pathways in various cancer types. Peaks on the curve indicate positive regulation, while downslopes indicate negative regulation. (B) The GSVA analyses using GO and KEGG datasets were performed, with results shown for BRCA, KIRC and LIHC. The blue bars represent the most significantly positively correlated pathways, the green bars represent the most significantly negatively correlated pathways and the grey bars represent non‐significant correlations (FDR > 0.05). The x‐axis represents the −log10 (*p*‐value) of the GSVA scores.

Through functional enrichment analysis of gene sequences, we explored the functional role of GMIP in tumours. Based on GMIP expression levels in different cancer types, we divided the samples into low and high expression groups and conducted GSEA and GSVA analyses to reveal the biological processes associated with GMIP. We demonstrated that GMIP plays a positive regulatory role in various immune‐related activities in BRCA, KIRC and LIHC and that KEGG pathways are significantly associated with GMIP. In BRCA and LIHC, GMIP is predicted to act as a negative regulator of protein synthesis and metabolism‐related pathways, while being a positive regulator of biological processes such as B cell activation, antigen presentation and the complement system. In KIRP, GMIP is also predicted to positively regulate B cell‐mediated immune responses and antigen processing and presentation (Figure 7A). To further investigate the biological significance of GMIP in these tumours, we performed GSVA analysis. The top 15 pathways significantly positively or negatively correlated with GMIP expression are shown in Figure 7B. The results indicate that GMIP expression is significantly associated with various immune‐related pathways, such as natural killer (NK) cell activation and regulation, T cell activation and regulation, interleukin signalling pathways (e.g., IL‐2 and IL‐4), tumour necrosis factor (TNF) regulation, neutrophil function and antigen processing and presentation. These findings suggest a potential link between GMIP expression and immune activation in the TME.

### 3.8 Silencing GMIP Causes Cell Proliferation and Migration

**FIGURE 8:**
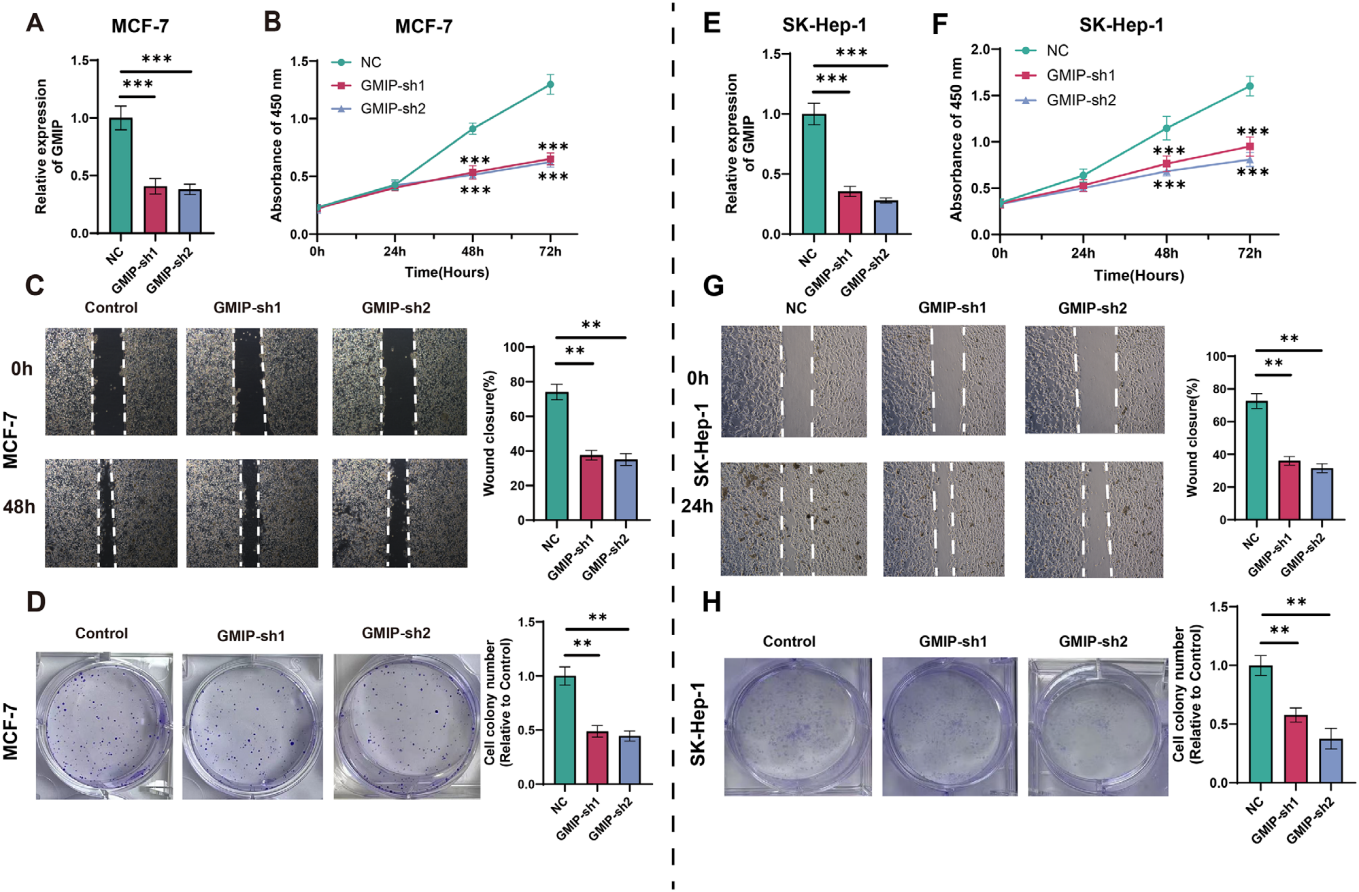
The silencing of GMIP leads to cell proliferation and migration. (A) The relative expression of GMIP in GMIP‐knockdown MCF‐7 cancer cells and SK‐Hep‐1 cancer cells, including GMIP‐sh1, GMIP‐sh2 and NC groups. (B) The CCK‐8 assay shows that the knockdown of GMIP significantly inhibits the proliferation of breast cancer cells. (C) Wound healing assay conducted on GMIP‐sh1, GMIP‐sh2 and NC group cancer cells. (D) Comparative analysis of colony formation between GMIP‐sh1, GMIP‐sh2 and control cell lines.

To investigate the relationship between GMIP expression and potential therapeutic agents, a comprehensive drug analysis was conducted (Figure S4). To explore GMIP's biological function and mechanism, we knocked down GMIP in the MCF‐7 cancer cell line and SK‐Hep‐1 cell line using siRNA. The results of the CCK‐8 and colony formation assays (Figure 8B,C) showed that GMIP knockdown significantly inhibited the proliferation of breast cancer cells. In addition, the reduction in GMIP expression also markedly weakened the migration ability of cancer cells (Figure 8D).

## 4 Discussion

Immunotherapy, particularly immune checkpoint blockade [37], has revolutionised cancer treatment. However, due to tumour microenvironment heterogeneity, only a subset of patients responds well [38]. Therefore, reliable biomarkers to predict responses are critical for personalised immunotherapy. In this study, we identified GMIP as a robust pan‐cancer prognostic biomarker, capable of predicting immunotherapy outcomes. Our findings also offer insights into GMIP's potential role in cancer immunity and its implications for future immunotherapy strategies [33].

To investigate GMIP's cellular mechanisms, we performed a comprehensive analysis using TCGA, HPA, CCLE, GTEx and TISCH databases, focusing on gene expression, survival prognosis, genetic variations and immune infiltration. Special attention was given to GMIP's genomic alterations across cancers and their prognostic significance, including its associations with CNV, DNA methylation, MSI, TMB and tumour immune infiltration.

We first analysed GMIP expression across various cancers using TCGA and GTEx data. GMIP was significantly downregulated in ACC, DLBC and THYM, but upregulated in most other cancer types. This contrasts with the traditional view that GMIP is suppressed in aggressive tumours and suggests it may have diverse roles depending on the cancer type.

Subsequently, we analysed the prognosis of cancer patients, including OS, DSS, DFI and PFS. The results indicate that high GMIP expression is negatively correlated with a favourable prognosis, which is consistent with conclusions drawn from previous studies on related family genes. The Rho pathway involving GMIP is highly associated with cancer, where RhoGAP activity is critical for the tumour‐suppressive function mediated by GMIP. Research has shown that DLC1, as a gene from the same family as GMIP, acts as a tumour suppressor in hepatocellular carcinoma and provides evidence supporting the hypothesis that DLC1 inhibits cancer cell growth by negatively regulating Rho protein activity [12, 17]. These findings underscore the importance of GMIP in predicting cancer patient survival and affirm its reliability as a prognostic indicator.

We analysed cancer prognosis, including OS, DSS, DFI and PFS. High GMIP expression was negatively correlated with favourable prognosis, consistent with previous findings on related family genes. GMIP's role in the Rho pathway, particularly its RhoGAP activity, is crucial for its tumour‐suppressive function. Similar to GMIP, DLC1—another family member—acts as a tumour suppressor in hepatocellular carcinoma by regulating Rho protein activity [12, 17]. These results highlight GMIP's value as a prognostic marker in cancer.

GMIP exhibits significant associations with TMB in seven cancer types and MSI in five, indicating its potential role in modulating immune checkpoint inhibitor response. Future research should investigate GMIP's regulatory role in the tumour microenvironment and MSI, as well as its potential to predict immunotherapy efficacy, enabling stratification for patients receiving checkpoint blockade therapy. Analysis of two independent cohorts confirmed that GMIP can predict sensitivity to immune checkpoint inhibitors (e.g., anti‐PD‐L1 and anti‐PD‐1 antibodies) [39]. Monitoring GMIP expression could help guide personalised treatment plans and improve the outcomes of conventional immunotherapy.

Our findings underscore GMIP's pivotal role in tumour immune regulation. The TME predicts not only tumour response to immunotherapy but also influences clinical outcomes [40, 41]. Normally, the immune system detects and eliminates tumour cells; however, tumours evade immune surveillance through various strategies. ESTIMATE analysis revealed that GMIP expression in the TME of 33 tumours is negatively correlated with stromal, immune and ESTIMATE scores, indicating its key role in TME regulation. High GMIP expression appears to be linked to a suppressive TME and weakened immune responses, potentially promoting tumour escape and progression.

Using the cBioPortal database, we found that GMIP mutations are more frequent in UCEC patients, with the strongest correlation between GMIP mutations and survival indicators observed in ACC patients. Data from the GEO database also link high GMIP expression to poor prognosis in BRCA, suggesting it as a negative prognostic factor. GSEA analysis revealed that high GMIP expression is enriched in immune regulatory processes, including T cell activation, TNF regulation, neutrophil function and antigen processing and presentation—key pathways involved in tumour cell stress response, repair and cell cycle regulation. Supporting our findings, Yvona Ward's research demonstrated that the GTP‐binding proteins Gem, negative regulators of the Rho‐Rho kinase pathway, play essential roles in BRCA prognosis, immune infiltration and immunotherapy response [42], suggesting that GMIP may impact the tumour immune environment through similar mechanisms. GSVA analysis further confirmed GMIP's involvement in immune‐related pathways across different cancer types, offering new insights into its role as an immune modulator. These findings not only uncover GMIP's potential mechanisms but also provide valuable clues for future cancer immunotherapy strategies targeting GMIP.

In follow‐up research, we identified potential anticancer drugs and explored their antitumour effects by modulating GMIP. Drug screening and sensitivity analysis based on GMIP expression revealed that docetaxel has strong potential as a candidate for molecular‐targeted therapy [43, 44]. Docetaxel, widely used in breast, lymphoma, lung and ovarian cancers, docetaxel primarily inhibits cancer cell growth by interfering with DNA replication and transcription and induces apoptosis by preventing DNA strand elongation [45, 46]. Additionally, it may enhance its effects by regulating enzyme activity and promoting M1 macrophage differentiation [47]. We hypothesise that part of its efficacy may result from targeting GMIP. Our study further suggests that GMIP‐targeted strategies could present new opportunities for drug development in BRCA‐deficient cancers and LIHC‐deficient cancers.

Our experimental findings demonstrated that GMIP knockdown significantly inhibited breast cancer cell proliferation and migration, suggesting its oncogenic potential. This leads to an intriguing future research direction: identifying and validating miRNAs that could naturally suppress GMIP expression. Recent advances in miRNA research have documented over 55,000 studies in PubMed, highlighting their crucial role in gene regulation and disease progression [48]. Our established analytical framework for GMIP could be extended to predict potential GMIP‐targeting miRNAs by integrating multiple data sources, including expression profiles, survival outcomes and immune infiltration patterns. The emergence of advanced computational models combining data fusion and model fusion approaches [49, 50] could facilitate the identification of novel miRNA–GMIP regulatory relationships. Furthermore, the integration of heterogeneous networks and contrastive learning techniques [51, 52] could help predict the complex interactions between GMIP and disease progression. This multidimensional analysis approach could reveal new therapeutic strategies where specific miRNAs could be employed to modulate GMIP expression, potentially enhancing the efficacy of cancer treatments. In conclusion, GMIP exerts a broad and profound influence across multiple cancers. Its expression is closely linked to immune regulatory factors, immune cell infiltration, the tumour microenvironment, TMB and MSI, highlighting its multifaceted role in tumour immune regulation [53]. This pan‐cancer analysis reveals GMIP's critical functions at various stages of tumorigenesis and progression. As research into GMIP and its regulatory network progresses, we anticipate breakthroughs in cancer diagnosis, treatment and prevention, fostering more targeted solutions and advancing the development of precision medicine and personalised therapy.

## Author Contributions

**Chao Jiang:** conceptualization (equal), data curation (equal), visualization (equal), writing – original draft (equal), writing – review and editing (equal). **Ningfeng Zhou:** data curation (equal), writing – original draft (equal), writing – review and editing (equal). **Xin Xu:** data curation (equal), writing – original draft (equal), writing – review and editing (equal). **Aochen Lv:** data curation (equal), methodology (equal), visualization (equal). **Shenren Chang:** data curation (equal), methodology (equal), visualization (equal). **Jiajie Wu:** methodology (equal), visualization (equal). **Xiang Li:** data curation (equal), methodology (equal), visualization (equal). **Aijun Sun:** conceptualization (equal), funding acquisition (equal), writing – review and editing (equal). **Shiyan Wang:** conceptualization (equal), funding acquisition (equal), writing – review and editing (equal). **WenZe Tian:** conceptualization (equal), funding acquisition (equal), writing – review and editing (equal).

## Conflicts of Interest

The authors declare no conflicts of interest.

## Supporting information


**Figure S1.** Schematic diagram of study design this study analysed mRNA expression profiles, somatic mutations and clinical data from multiple databases. We examined GMIP’s differential expression in cancerous and non‐cancerous tissues, as well as in various cell types. Cox regression analysis, based on optimal survival split points, revealed that GMIP expression is linked to genomic instability, as shown by data from cBioPortal and GSCA. We also assessed the clinical relevance of abnormal CNV and methylation, and compared GMIP expression with TMB and MSI across cancers. The relationship between GMIP expression and ESTIMATE scores, immune cell infiltration and immune‐related genes was visualised. Functional annotation analysis further explored GMIP’s role in cancer immunity. Additionally, GMIP‐related chemotherapy responses were predicted, potential drugs were identified via molecular docking, and experimental validation was performed.

**Figure S2.** Univariate Cox regression analysis of GMIP expression across pan‐cancer tissues. The forest plot illustrates the relationship between GMIP expression and OS, DFS, DSS and PFS in pan‐cancer patients.

**Figure S3.** GMIP expression was correlation with immune response genes, including (A) MHC genes, (B) immunosuppressive genes, (C) Chemokines receptors, (D) Immune activation genes and (E) Chemokines.

**Figure S4.** The relationship between GMIP expression and drug sensitivity, and molecular docking of GMIP‐targeted compounds. (A) The relationship between GMIP expression and predicted drug response. (B) The band structure of GMIP protein and the stick representation of docetaxel. (C) A close‐up view of the interaction between docetaxel and the GMIP protein, with important receptor residues represented by sticks. Docetaxel is shown in blue, and receptor residues involved in ligand binding are shown in green.

Tables S1–S3.

## Data Availability

The original contributions presented in the study are included in the article materials. Further inquiries can be directed to the corresponding author.
