# Supplementary material for: GMIP: A Novel Prognostic Biomarker Influencing Immune Infiltration and Tumour Dynamics Across Cancer Types
Source: J Cell Mol Med. 2025 Apr 24;29(8):e70476. doi: 10.1111/jcmm.70476 (PMC12021672; doi:10.1111/jcmm.70476)
Supplement: Supplementary file 1 — Figure S1. Schematic diagram of study design this study analysed mRNA expression profiles, somatic mutations and clinical data from multiple databases. We examined GMIP’s differential expression in cancerous and non‐cancerous tissues, as well as in various cell types. Cox regression analysis, based on optimal survival split points, revealed that GMIP expression is linked to genomic instability, as shown by data from cBioPortal and GSCA. We also assessed the clinical relevance of abnormal CNV and methylation, and compared GMIP expression with TMB and MSI across cancers. The relationship between GMIP expression and ESTIMATE scores, immune cell infiltration and immune‐related genes was visualised. Functional annotation analysis further explored GMIP’s role in cancer immunity. Additionally, GMIP‐related chemotherapy responses were predicted, potential drugs were identified via molecular docking, and experimental validation was performed. [file JCMM-29-e70476-s005.pdf]

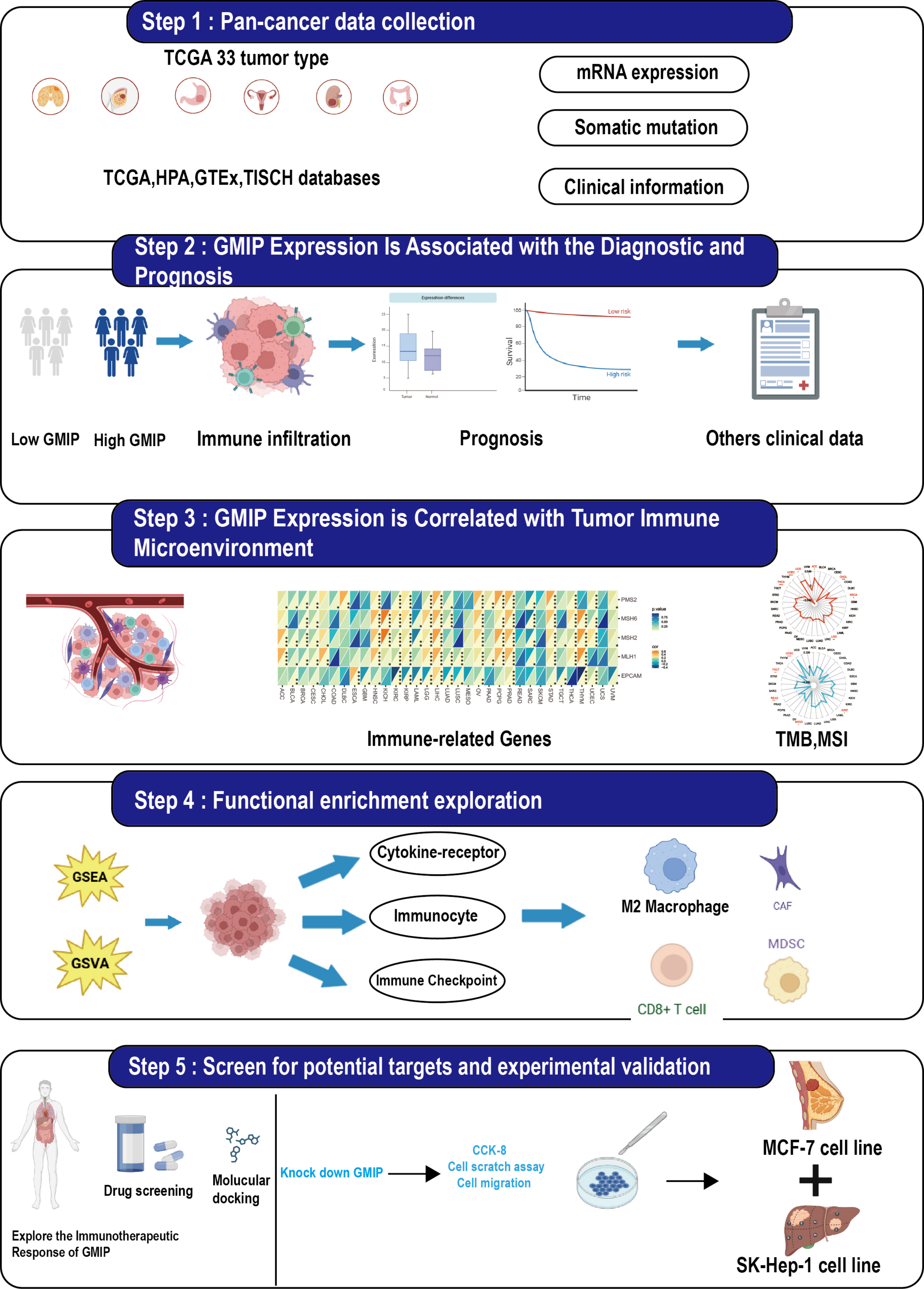

Step 1 : Pan-cancer data collection
TCGA 33 tumor type
TCGA,HPA,GTEx,TISCH databases
mRNA expression
Somatic mutation
Clinical information
Step 2 : GMIP Expression Is Associated with the Diagnostic and Prognosis
Low GMIP
High GMIP
Immune infiltration
Prognosis
Others clinical data
Step 3 : GMIP Expression is Correlated with Tumor Immune Microenvironment
Immune-related Genes
TMB,MSI
Step 4 : Functional enrichment exploration
GSEA
GSVA
Cytokine-receptor
Immunocyte
Immune Checkpoint
M2 Macrophage
CAF
MDSC
CD8+ T cell
Step 5 : Screen for potential targets and experimental validation
Drug screening
Molucular docking
Explore the Immunotherapeutic Response of GMIP
Knock down GMIP
CCK-8
Cell scratch assay
Cell migration
MCF-7 cell line
SK-Hep-1 cell line
