# Supplementary figures and images for: GMIP: A Novel Prognostic Biomarker Influencing Immune Infiltration and Tumour Dynamics Across Cancer Types
Source: J Cell Mol Med. 2025 Apr 24;29(8):e70476. doi: 10.1111/jcmm.70476 (PMC12021672; doi:10.1111/jcmm.70476)

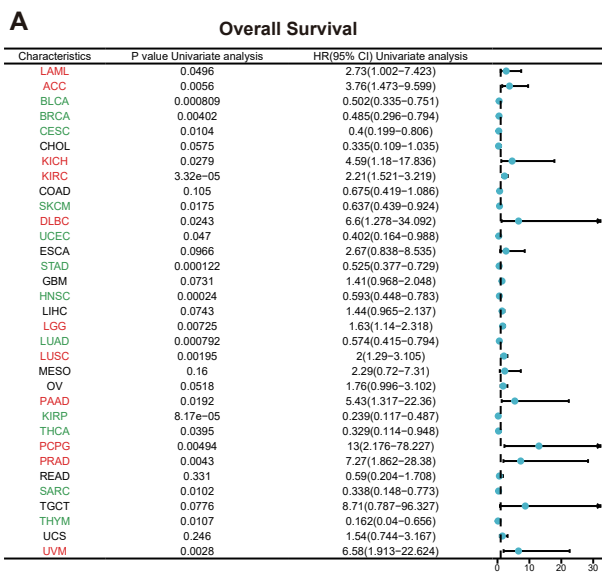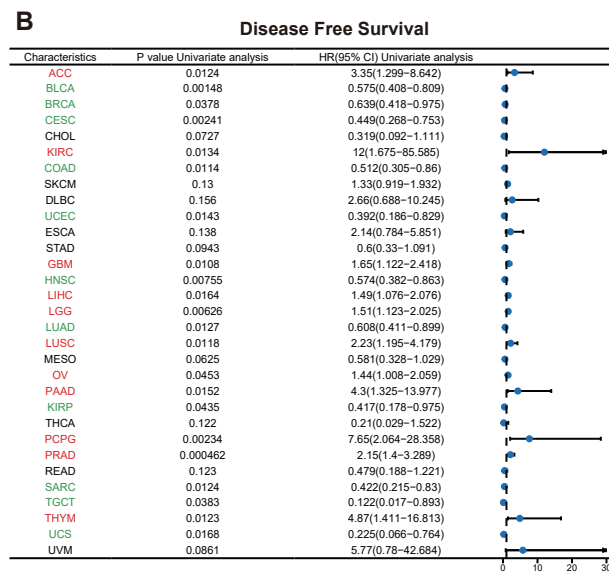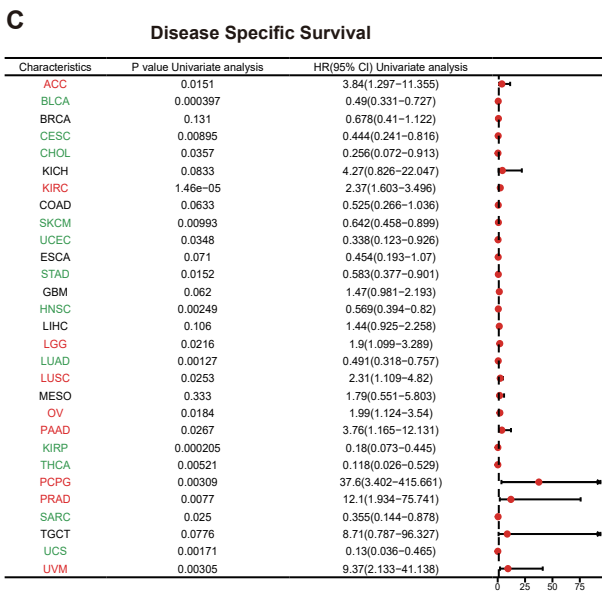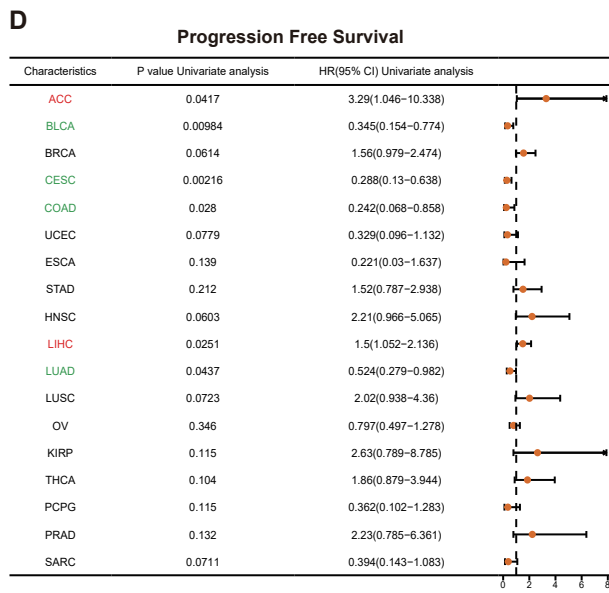

Supplement: Supplementary file 2 — Figure S2. Univariate Cox regression analysis of GMIP expression across pan‐cancer tissues. The forest plot illustrates the relationship between GMIP expression and OS, DFS, DSS and PFS in pan‐cancer patients. [file JCMM-29-e70476-s002.pdf]

A

GMIP Bubble Chart of Spearman Correlation and P-value

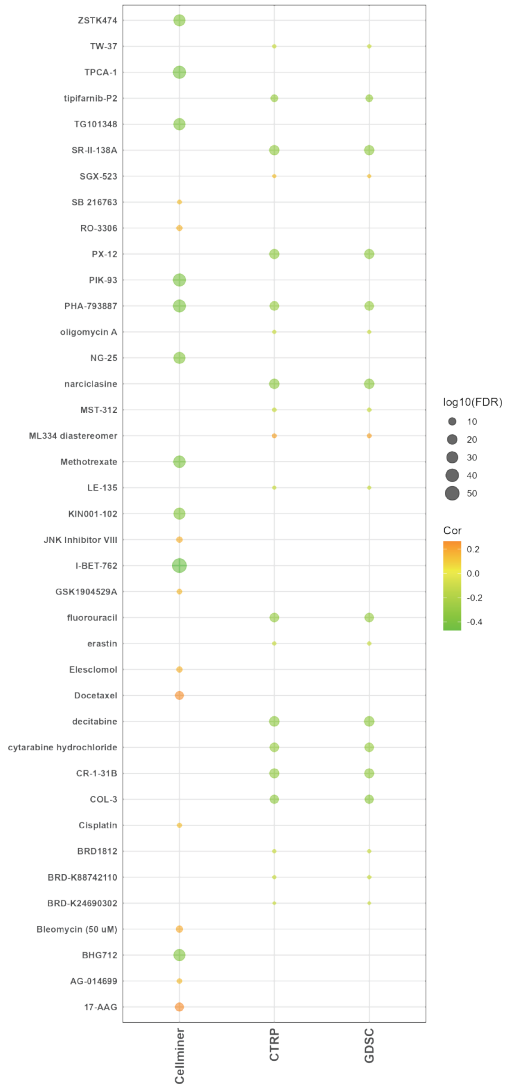

B

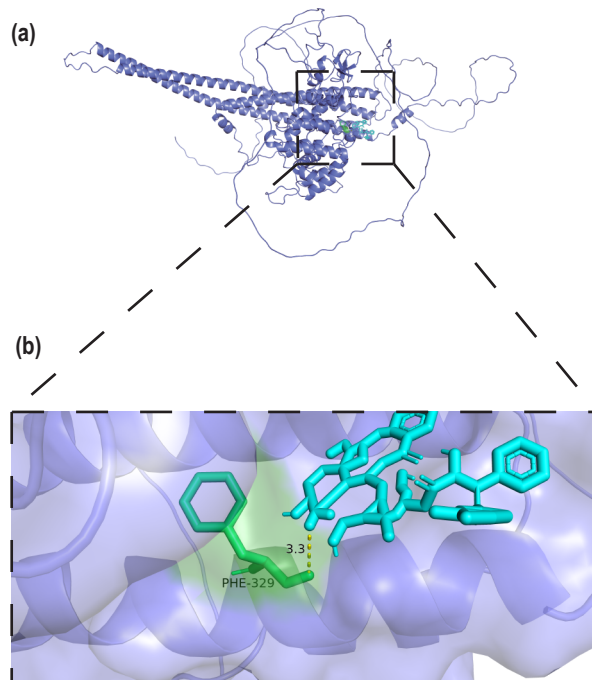

Supplement: Supplementary file 4 — Figure S4. The relationship between GMIP expression and drug sensitivity, and molecular docking of GMIP‐targeted compounds. (A) The relationship between GMIP expression and predicted drug response. (B) The band structure of GMIP protein and the stick representation of docetaxel. (C) A close‐up view of the interaction between docetaxel and the GMIP protein, with important receptor residues represented by sticks. Docetaxel is shown in blue, and receptor residues involved in ligand binding are shown in green. [file JCMM-29-e70476-s001.pdf]
