# Supplementary material for: GMIP: A Novel Prognostic Biomarker Influencing Immune Infiltration and Tumour Dynamics Across Cancer Types
Source: J Cell Mol Med. 2025 Apr 24;29(8):e70476. doi: 10.1111/jcmm.70476 (PMC12021672; doi:10.1111/jcmm.70476)
Supplement: Supplementary file 3 — Figure S3. GMIP expression was correlation with immune response genes, including (A) MHC genes, (B) immunosuppressive genes, (C) Chemokines receptors, (D) Immune activation genes and (E) Chemokines. [file JCMM-29-e70476-s004.pdf]

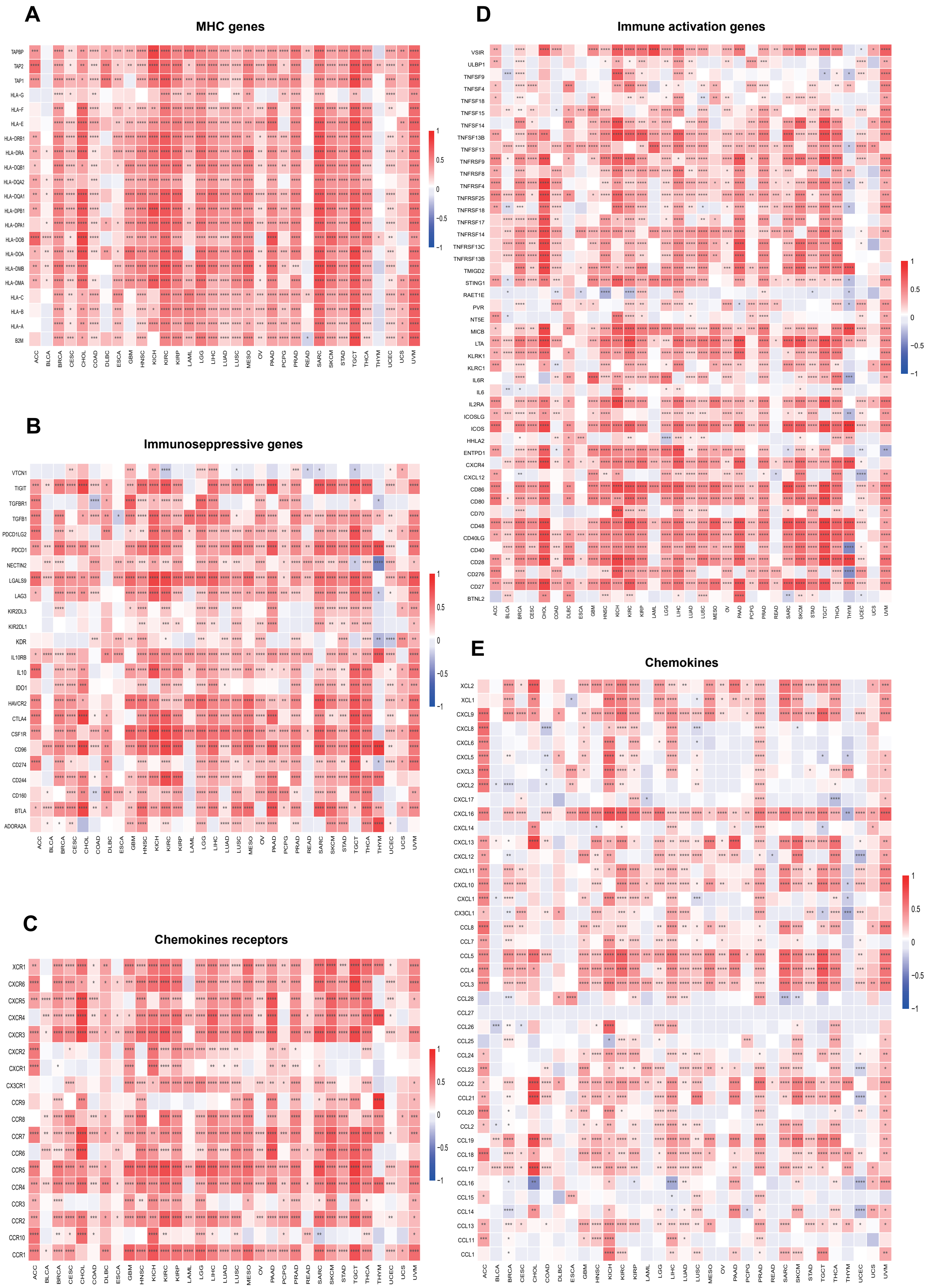
A
MHC genes
TAPBP
TAP2
TAP1
HLA-G
HLA-F
HLA-E
HLA-DRB1
HLA-DRA
HLA-DQB1
HLA-DQA2
HLA-DQA1
HLA-DPB1
HLA-DPA1
HLA-DOB
HLA-DOA
HLA-DMB
HLA-DMA
HLA-C
HLA-B
HLA-A
B2M
ACC BLCA BRCA CESC CHOL COAD DLBC ESCA GBM HNSC KICH KIRC KIRP LAML LGG LIHC LUAD LUSC MESO OV PAAD PCPG PRAD READ SARC SKCM STAD TGCT THCA THYM UCEC UCS UVM
1 0.5 0 -0.5 -1
B
Immunoseppressive genes
VTCN1
TIGIT
TGFBR1
TGFB1
PDCD1LG2
PDCD1
NECTIN2
LGALS9
LAG3
KIR2DL3
KIR2DL1
KDR
IL10RB
IL10
IDO1
HAVCR2
CTLA4
CSF1R
CD96
CD274
CD244
CD160
BTLA
ADORA2A
C
Chemokines receptors
XCR1
CXCR6
CXCR5
CXCR4
CXCR3
CXCR2
CXCR1
CX3CR1
CCR9
CCR8
CCR7
CCR6
CCR5
CCR4
CCR3
CCR2
CCR10
CCR1
D
Immune activation genes
VSIR
ULBP1
TNFSF9
TNFSF4
TNFSF18
TNFSF15
TNFSF14
TNFSF13B
TNFSF13
TNFRSF9
TNFRSF8
TNFRSF4
TNFRSF25
TNFRSF18
TNFRSF17
TNFRSF14
TNFRSF13C
TNFRSF13B
TMIGD2
STING1
RAET1E
PVR
NT5E
MICB
LTA
KLRK1
KLRC1
IL6R
IL6
IL2RA
ICOSLG
ICOS
HHLA2
ENTPD1
CXCR4
CXCL12
CD86
CD80
CD70
CD48
CD40LG
CD40
CD28
CD276
CD27
BTNL2
E
Chemokines
XCL2
XCL1
CXCL9
CXCL8
CXCL6
CXCL5
CXCL3
CXCL2
CXCL17
CXCL16
CXCL14
CXCL13
CXCL12
CXCL11
CXCL10
CXCL1
CX3CL1
CCL8
CCL7
CCL5
CCL4
CCL3
CCL28
CCL27
CCL26
CCL25
CCL24
CCL23
CCL22
CCL21
CCL20
CCL2
CCL19
CCL18
CCL17
CCL16
CCL15
CCL14
CCL13
CCL11
CCL1
