# Supplementary material for: GMIP: A Novel Prognostic Biomarker Influencing Immune Infiltration and Tumour Dynamics Across Cancer Types
Source: J Cell Mol Med. 2025 Apr 24;29(8):e70476. doi: 10.1111/jcmm.70476 (PMC12021672; doi:10.1111/jcmm.70476)
Supplement: Supplementary file 5 — Tables S1–S3. [file JCMM-29-e70476-s003.docx]

**SUPPLEMENTAL MATERIALS**

**SUPPLEMENTAL TABLE**

**TABLE S1 TCGA cancer abbreviations and the corresponding cancer type**

| Abbreviations | Cancer Type |
| --- | --- |
| ACC | Adrenocortical carcinoma |
| BLCA | Bladder Urothelial Carcinoma |
| BRCA | Breast invasive carcinoma |
| CESC | Cervical squamous cell carcinoma and endocervical adenocarcinoma |
| CHOL | Cholangiocarcinoma |
| COAD | Colon adenocarcinoma |
| DLBC | Lymphoid Neoplasm Diffuse Large B- cell Lymphoma |
| ESCA | Esophageal carcinoma |
| GBM | Glioblastoma multiforme |
| HNSC | Head and Neck squamous cell carcinoma |
| KICH | Kidney Chromophobe |
| KIRC | Kidney renal clear cell carcinoma |
| KIRP | Kidney renal papillary cell carcinoma |
| LAML | Acute Myeloid Leukemia |
| LGG | Brain Lower Grade Glioma |
| LIHC | Liver hepatocellular carcinoma |
| LUAD | Lung adenocarcinoma |
| LUSC | Lung squamous cell carcinoma |
| MESO | Mesothelioma |
| OV | Ovarian serous cystadenocarcinoma |
| PAAD | Pancreatic adenocarcinoma |
| PCPG | Pheochromocytoma and Paraganglioma |
| PRAD | Prostate adenocarcinoma |
| READ | Rectum adenocarcinoma |
| SARC | Sarcoma |
| SKCM | Skin Cutaneous Melanoma |
| STAD | Stomach adenocarcinoma |
| TGCT | Testicular Germ Cell Tumors |
| THCA | Thyroid carcinoma |
| THYM | Thymoma |
| UCEC | Uterine Corpus Endometrial Carcinoma |
| UCS | Uterine Carcinosarcoma |
| UVM | Uveal Melanoma |

**Table S2.** **siRNA sequences of NC and GMIP used in our experiments**

|  | sense（5'-3'） | antisense（5'-3'） |
| --- | --- | --- |
| si-NC | UUCUCCGAACGUGUCACGUTT | ACGUGACACGUUCGGAGAATT |
| si- GMIP #1 | CCAGAUUGUGAAGACGGACCCAUAU | AUAUGGGUCCGUCUUCACAAUCUGG |
| si- GMIP #2 | CAGAUUGUGAAGACGGACCCAUAUA | UAUAUGGGUCCGUCUUCACAAUCUG |

**Table S3.** **Primer sequence of β-actin and GMIP used in our experiments**

| Gene name | Forward primer，5’-3’ | Reverse primer，3’-5’ |
| --- | --- | --- |
| β-actin (Human) | GGCATCCTCACCCTGAAGTACC | CCACACGCAGCTCATTGTAGAAG |
| GMIP(Human) | CACCCTGTTTCTGGAGCACGATC | CCTTCCGCCACTTCTCAATCTCAG |

**SUPPLEMENTARY FIGURE CAPTIONS**

SUPPLEMENTARY FIGURE 1 | Schematic Diagram of Study Design This study analyzed mRNA expression profiles, somatic mutations, and clinical data from multiple databases. We examined GMIP's differential expression in cancerous and non-cancerous tissues, as well as in various cell types. Cox regression analysis, based on optimal survival split points, revealed that GMIP expression is linked to genomic instability, as shown by data from cBioPortal and GSCA. We also assessed the clinical relevance of abnormal CNV and methylation, and compared GMIP expression with TMB and MSI across cancers. The relationship between GMIP expression and ESTIMATE scores, immune cell infiltration, and immune-related genes was visualized. Functional annotation analysis further explored GMIP’s role in cancer immunity. Additionally, GMIP-related chemotherapy responses were predicted, potential drugs were identified via molecular docking, and experimental validation was performed.

SUPPLEMENTARY FIGURE 2 | Univariate Cox regression analysis of GMIP expression across pan-cancer tissues. The forest plot illustrates the relationship between GMIP expression and OS, DFS, DSS, and PFS in pan-cancer patients.

SUPPLEMENTARY FIGURE 3 | GMIP expression was correlation with immune response genes, including (A) MHC genes, (B) immunoseppressive genes, (C) Chemokines receptors, (D) Immune activation genes, (E) Chemokines.

SUPPLEMENTARY FIGURE 4 | The relationship between GMIP expression and drug sensitivity, and molecular docking of GMIP-targeted compounds. (A) The relationship between GMIP expression and predicted drug response. (B) The band structure of GMIP protein and the stick representation of docetaxel. (C) A close-up view of the interaction between docetaxel and the GMIP protein, with important receptor residues represented by sticks. Docetaxel is shown in blue, and receptor residues involved in ligand binding are shown in green.
